# Plant Fibers as Composite Reinforcements for Biomedical Applications

**DOI:** 10.3390/bioengineering10070804

**Published:** 2023-07-05

**Authors:** Lizbeth Zamora-Mendoza, Fernando Gushque, Sabrina Yanez, Nicole Jara, José F. Álvarez-Barreto, Camilo Zamora-Ledezma, Si Amar Dahoumane, Frank Alexis

**Affiliations:** 1 Departamento de Ingeniería Química, Colegio de Ciencias e Ingenierías, Instituto de Microbiología, Institute for Energy and Materials, Universidad San Francisco de Quito USFQ, Quito 170901, Ecuador; lzamora@estud.usfq.edu.ec (L.Z.-M.); jalvarezb@usfq.edu.ec (J.F.Á.-B.); 2 School of Biological Sciences and Engineering, Yachay Tech University, Urcuquí 100119, Ecuador; egushque@yachaytech.edu.ec (F.G.); sabrina.yanez@yachaytech.edu.ec (S.Y.); nicole.jara@yachaytech.edu.ec (N.J.); 3 Green and Innovative Technologies for Food, Environment and Bioengineering Research Group (FEnBeT), Faculty of Pharmacy and Nutrition, UCAM-Universidad Católica de Murcia, Avda, Los Jerónimos 135, Guadalupe de Maciascoque, 30107 Murcia, Spain; czamora9@ucam.edu; 4 Department of Chemistry and Biochemistry, Université de Moncton, 18 Avenue Antonine-Maillet, Moncton, NB E1A 3E9, Canada

**Keywords:** plant, fibers, composites, biomaterials, polymers, biomedical applications

## Abstract

Plant fibers possess high strength, high fracture toughness and elasticity, and have proven useful because of their diversity, versatility, renewability, and sustainability. For biomedical applications, these natural fibers have been used as reinforcement for biocomposites to infer these hybrid biomaterials mechanical characteristics, such as stiffness, strength, and durability. The reinforced hybrid composites have been tested in structural and semi-structural biodevices for potential applications in orthopedics, prosthesis, tissue engineering, and wound dressings. This review introduces plant fibers, their properties and factors impacting them, in addition to their applications. Then, it discusses different methodologies used to prepare hybrid composites based on these widespread, renewable fibers and the unique properties that the obtained biomaterials possess. It also examines several examples of hybrid composites and their biomedical applications. Finally, the findings are summed up and some thoughts for future developments are provided. Overall, the focus of the present review lies in analyzing the design, requirements, and performance, and future developments of hybrid composites based on plant fibers.

## 1. Introduction

**Figure 1:**
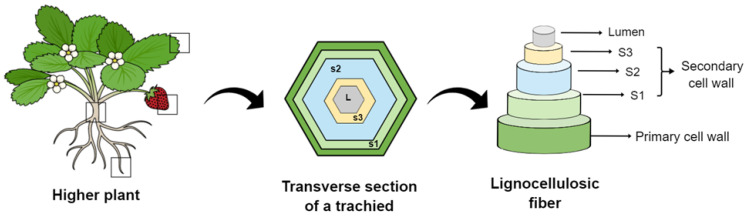
General schematic of plant cell-wall layers and lignocellulosic fibers that can be extracted from different parts of plants, such as the leaves, fruits, stems, and roots.

Fiber-reinforced composites have been gradually developed over the past few decades to provide them unique characteristics and properties [1]. Plant fibers are obtained from different plant parts, such as the stem, root, leaf, and fruit [2]. They are classified as wood or non-wood (such as bast) fibers [3]. Most plant fibers, except for cotton, comprise cellulose, hemicellulose, lignin, waxes, and some water-soluble compounds [4]. They are formed by thin strands of semicrystalline cellulose coated with an amorphous layer of pectin and hemicellulose [5]. Each plant fiber is a single cell ranging from 1–50 mm to 10–50 µm in diameter. Structurally, a plant fiber is surrounded by a cellular wall with plenty of fibrils and an inner secondary wall composed of the following three layers: S1, S2, and S3, as seen in Figure 1 [6]. The S2 layer has a series of helical cellulose microfibrils embedded in a soft matrix of hemicellulose and lignin [7]. Despite this complex hierarchical structure, the S2 layer is the most important layer in terms of mechanical properties of a single fiber due to its thickness and ultrastructure [8]. The chemical composition and properties of plant-based natural fibers differ significantly according to the plant section from which they are extracted [9].

**Figure 2:**
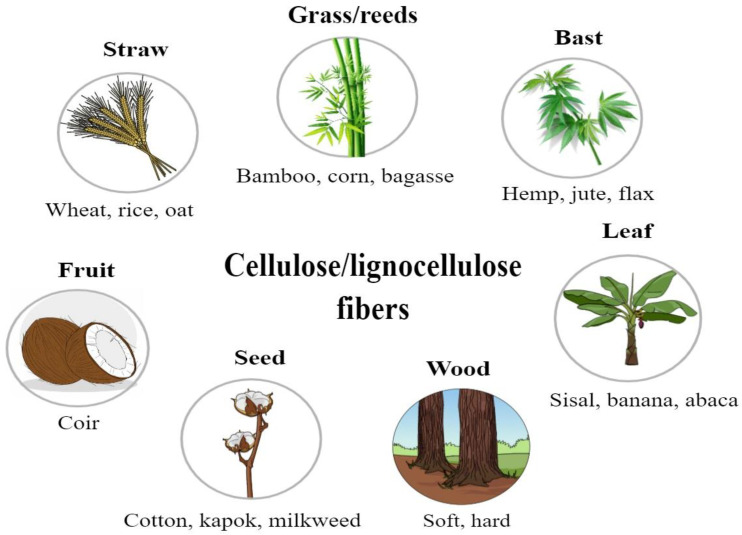
Different parts of higher plants used to extract cellulose/lignocellulose fibers.

The extraction process of plant fibers involves several steps that start with harvesting the plant material. Fibers are then separated from the non-cellulosic material using a method such as retting or pulping. Extraction is conducted through either chemical or mechanical methods. First, it breaks down the pectin, lignin, and hemicellulose that bind the fibers. Once separated, fibers are cleaned to remove any impurities and, subsequently, dried. Specific methods in the extraction process vary depending on the source and intended use of the fibers. Common sources of plant fibers include seeds and fruit (e.g., cotton, kapok, and coir), bast and leaves (e.g., sisal, jute, flax, and hemp), straw (e.g., rice, corn, and wheat), and wood (e.g., softwood and hardwood) [10,11,12] (Figure 2).

Plant fibers are attractive for many industrial applications requiring unique mechanical properties owing to their special features, such as low cost, low density, flexibility, tensile strength, elasticity, rigidity, non-toxicity, and biodegradability [13,14,15]. Lower prices of natural fibers are related to their easy manufacture process and the fact that they can be obtained from renewable sources [16]. In particular, they have witnessed novel advances in the biomedical field for applications in tissue engineering and wound dressing due to their biocompatibility with mucous membranes, high water-binding affinity, and swelling capacity [17,18]. Other examples include the design of drug-loaded cellulose-based bandages [19]. Notably, using plant fibers in biomedical devices is important to improve biodegradability and reduce immunogenicity [20,21,22,23,24].

Incorporating plant fibers into various applications can reduce the industrial reliance on landfills and elicit the development of the circular economy. The UNEP DTU Partnership and United Nations Environment Programme reported that, in the study titled Reducing Consumer Food Waste Using Green and Digital Technologies, around 931 million tons of food waste, including plant waste (fruit, vegetable, and cereals) are being discarded each year [25]. Consequently, a considerable amount of high-value compounds, such as nutrients, biomass, and bioactive components, is lost [26,27]. However, adequate processing technology makes it possible to recover and reuse discarded vegetables. Common waste includes soybean hulls, wheat straw, sugarcane, rice straw and husks, palm oil residue, pineapple, banana leaf fiber, bagasse, hemp, and flax straws [28,29]. Given that cellulose fibers can be isolated from plant waste, many industrial sectors are now interested in sustainable and eco-friendly products to promote the circular economy. Sustainability is mainly addressed using raw materials, operating supplies, ingredients, and high-value products from discarded materials, such as vegetable residues [30].

## 2. Importance of Composites

Ancient civilizations, such as the Egyptians, Greeks, and Romans, used natural composites made of straw, mud, and animal dung to build structures, such as houses and walls [31]. These early composites were not very strong, but still offered better insulation and durability than the individual materials used on their own [32,33]. Nowadays, composites are used in various fields, such as the automotive, aerospace, mechanical, marine and biomedical industries in addition to other sectors, namely chemistry, construction, ground transportation, and environmentally sustainable energy [34,35]. The industry of composites is estimated to exceed 100 billion dollars globally, making these outstanding materials one of the largest and most critical engineered materials after the steel industry [36].

**Figure 3:**
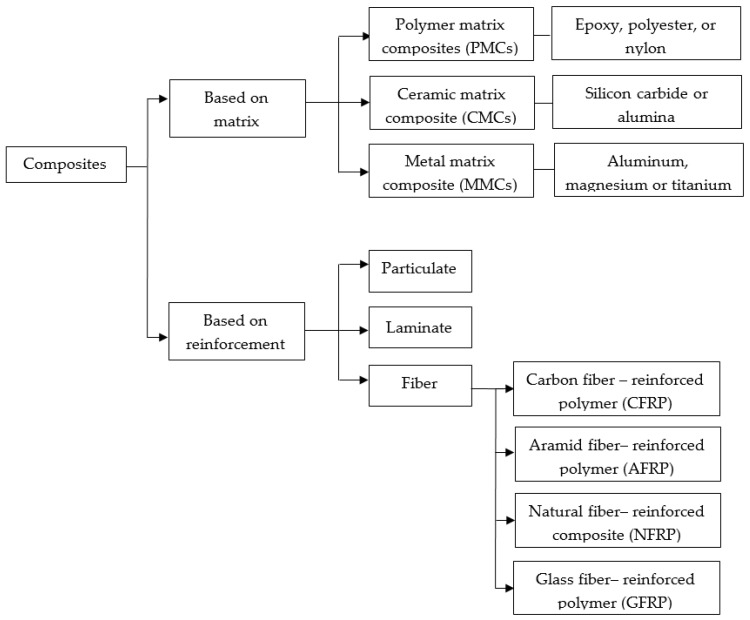
Classification of composites based on either the matrix or the reinforcement.

Composites are made up of two phases that fulfill specific functions. The first is a strong discontinuous phase called the reinforcement material; it is embedded in a secondary, more ductile, and less complex continuous phase called the matrix [37]. Matrices are usually made of metal, ceramic, and polymers, and the reinforcement can be fibrous (synthetic and natural), particulate, or laminate (Figure 3) [38,39]. New trends focus on using composites of synthetic materials, such as glass, carbon fibers, ceramics, and metal-based materials as reinforcement [40]. Common composites for biomedical applications are a mixture of hydroxyapatite/polyethylene, silica/silicone rubber, carbon fiber/ultra-high molecular weight polyethylene, carbon fiber/epoxy, and carbon fiber/polyetheretherketone [41]. In this sense, composites offer several advantages over other materials due to their physical and chemical characteristics. Compared to raw materials, composites have a better strength-to-weight ratio, higher tensile strength, superior torsional stiffness, and impact properties. Additionally, composites have a higher fatigue resistance limit and excellent corrosion resistance that make them suitable for load-bearing applications in orthopedics [42,43].

Overall, composite materials have shown great promise in the field of biomedical applications due to their unique combinations of properties that cannot be achieved with traditional materials. Carbon fiber-reinforced polymer (CFRP) composites, for instance, are used for prosthetic limbs and orthopedic implants because of their lightweight nature and strength [44]. Natural fiber-reinforced polymer composites made from bamboo, jute, or flax are used for surgical meshes and wound dressings because of their biocompatibility and high tensile strength [45]. Polymer–ceramic composites, such as hydroxyapatite-reinforced polyethylene, are used for bone substitutes and dental implants because of their biocompatibility and ability to cellular integration [46]. Finally, polymer–metal composites, such as titanium-reinforced polyetheretherketone (PEEK), are used for spinal implants and other orthopedic devices due to their high strength and fatigue resistance [47].

Currently, there is a pressing need for a new generation of composites that can combine both synthetic and natural materials, with the ultimate goal of creating entirely environmentally friendly materials [48]. Vegetable fiber-reinforced composites have been increasingly used in biomedical applications due to their biocompatibility, renewability, and potential for low-cost production [10]. By using these composites in biomedical applications, we can provide a promising alternative to traditional materials, such as metals and ceramics, which could lead to the development of more sustainable and environmentally friendly medical devices [49,50,51,52,53,54].

## 3. Plant Fibers

### 3.1. Current Plant Fibers Used in Biomedical Applications

Natural fibers used in medicine can be extracted from inexpensive and natural resources, such as bast fibers (jute, flax), seed fibers (cotton, milkweed), leaf fibers (sisal, agave), grass fibers (bamboo) and straw fibers (rice, corn) [10]. Certain natural biopolymers such as kemp, sisal, and cotton contain biomolecules related to antioxidant, antibacterial and antiproliferative activity [55]. To achieve optimal performance, natural fibers should include factors and inherent characteristics, including fiber quality, structure, and mechanical properties. Nevertheless, the temperature, humidity, altitude, and climatic conditions influence those characteristics [56]. On the one hand, fiber quality encompasses several factors, including fiber length, diameter, strength, flexibility, and fineness, which are critical factors in determining their processability and end-use applications. Longer fibers are desirable for applications requiring high tensile strength [57]. Fiber strength is another crucial quality parameter, as it determines the durability and load-bearing capacity of the final product. Moreover, the flexibility and fineness of fibers influence their handling and ease of manipulation during processing. On the other hand, the structure of plant fibers refers to their cellular arrangement, composition, and orientation; for instance, highly aligned microfibrils result in fibers with enhanced strength and stiffness [58]. Additionally, the presence of secondary walls and intercellular spaces within the fiber structure impacts its porosity, moisture absorption, and permeability.

According to Karimah et al., other factors, such as storage period and condition, can affect fiber qualities; thus, those elements should be monitored efficiently [28]. Similarly, the maturation time of the plant and processing method affects the surface properties and diameter length of the natural fibers. Additionally, the structural dimensions of the natural fibers are critical for industrial applications [59]. For this reason, biopolymers could require modifications or additives to improve their properties for engineering applications.

Plant-based natural fibers can be used for biomedical applications based on their composition, sustainable potential, and biological function [60]. Usually, natural fibers used in biomedicine have been modified through surface treatment, processing, or annealing with other materials [61]. The features and potential biomedical applications of natural fibers are presented in Table 1.

### 3.2. Mechanical and Chemical Properties of Plant Fibers

Various synthetic fibers are extensively used in the biomedical field due to their mechanical properties and versatility [75]. However, biodegradability is still a critical limitation for most of them [76]. In this context, scientists found it appropriate to use natural fibers to meet specific requirements from industry, such as low production cost, renewability, and sustainability [28]. Compared to many synthetic fibers, plant fibers present higher strength and stiffness depending on their chemical composition and polymeric structure [61]. In addition, natural fibers exert low immunogenicity and are lightweight, which makes them appropriate for cellular integration applications in implants [77]. Conversely, plant fibers show limited durability and poor moisture resistance due to their chemical structure (hydroxyl and polar groups) and degree of crystallinity [78].

Fundamentally, the physical and chemical composition of raw material play an important role in the final mechanical properties of natural fibers [74]. These also depend on the natural fiber size and the processing method, as well as the maturation stage of the plant [79]. The understanding of mechanical properties, such as stress resistance, production yield, fatigue, tensile strength, toughness, hardness, and brittleness, is essential in the medical field [39,80]. Table 2 summarizes some of the most essential mechanical properties of natural fibers for their use in medical applications.

**Figure 4:**
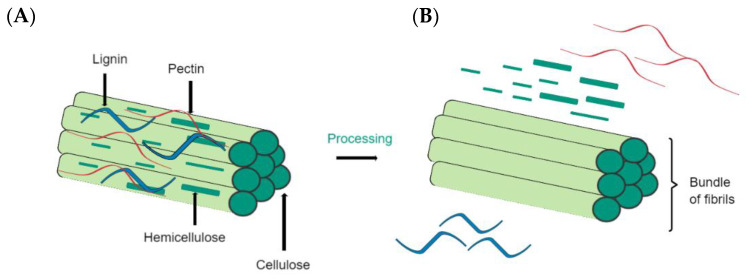
Main components of plant fibers. (**A**) Schematic of a simplified structure of plant fibers. (**B**) Fibrils of cellulose after treatment.

The main components of plant fibers are sugar-based polymers, such as cellulose, which is incorporated into a matrix containing hemicellulose, lignin, and pectin (see Figure 4). Cellulose is the main component of natural fibers and constitutes the glucose units linked by β-1,4-glycosidic bonds [9,84]. Cellulose has a semicrystalline form because it has crystalline and amorphous phases. Hemicellulose is the second most abundant component in natural plant fibers. Made of heteroglycan sugar units, it comprises a degree of polymerization (DP) of 150–200. In addition, hemicellulose can contain various proportions of mannose, galactose, pentose, xylose, fucose, and arabinose. Lastly, lignin is a non-crystalline molecule that is built of phenyl-propane units. Unlike cellulose, lignin is a three-dimensional polymer, is not vulnerable to hydrolysis, and is durable [85].

**Figure 5:**
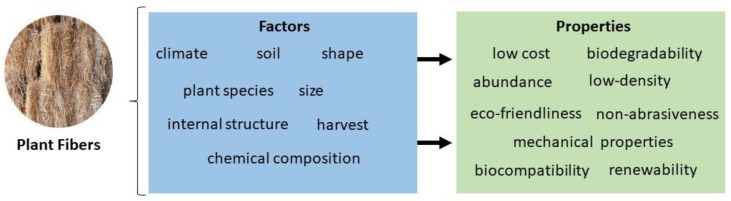
Factors affecting the properties of natural plant fibers.

Figure 5 regroups the different factors that affect the properties of plant fibers. Among these, the mechanical properties are of paramount importance. For instance, the tensile strength and Young’s modulus proportionally depend on the amount of cellulose the fiber contains [86]. However, the elongation at break, which refers to the resistance of plant fibers to change without causing cracks, increases if the cellulose content is low [14,87]. On the other hand, the increase in hemicellulose content can lead to a decrease in the tensile strength of the fiber since hemicellulose is characterized by having an amorphous and non-homogeneous structure [14]. Natural fibers have mechanical properties inferior to those observed in synthetic fibers [88]. However, plant fibers require less energy to produce and have properties of interest, such as high stiffness, resistance, and non-toxicity [89].

Plant fibers are characterized by their ability to absorb water because they have a high percentage of cellulose, which is a hydrophilic molecule. Therefore, the high cellulose content is reflected in the increased water absorption. This property is considered a valuable advantage over synthetic fibers, which are hydrophobic [90,91]. Another essential property of plant fibers is that they constitute biodegradable materials. The hemicellulose content plays a substantial role in biodegradability and moisture sorption, which means that increasing hemicellulose content increases the degradability of natural fibers. Natural fibers are degraded and transformed into CO_2_, H_2_O, hydrocarbons, methane, and biomass through chemical and biological reactions [92]. Table 3 summarizes the composition and mechanical properties of different fibers extracted from different plants.

As discussed above (Figure 4), the diversity in the properties of plant fibers results from different factors, such as climate, soil, species, harvest, etc. [10]. The fiber size and shape and extraction process can affect the quality of plant natural fibers [4]. In the same way, internal structure and chemical composition are responsible for density, electrical resistivity, and tensile strength [97]. These properties can be improved and optimized only by using the appropriate chemical treatments [58]. 

### 3.3. Preparation of Composites Based on Plant Fibers

Composites are composed of at least two different materials, separated by interphases, to obtain better properties [98]. The continuous and discontinuous phases of composites are known as matrix and reinforcement, respectively [99]. Biocomposites are composites that contain at least one natural support [100]. Plant/natural fibers are reinforcing materials in a matrix responsible for binding and protecting the fibers [101]. The desired properties of the composite will directly depend on the type and percentage of the matrix, natural fibers used, manufacturing method, and fiber orientation. Polymers (thermosets or thermoplastics) are commonly used as the matrix to support the plant fibers and hold the loads [102]. The reinforcement amount in the composite can vary depending on the type, fiber size, and desired properties. For instance, the optimal percentage of reinforcement can be between 20 and 50% of the composite [103]. Singha et al. prepared d unsaturated polyesters (UPE) reinforced using *Grewia optiva* fibers and determined the percentage to be 30% of the fiber loading to obtain optimal properties, such as tensile, flexural, and compressive strength [104]. Similar results were obtained by Ozturk et al. when synthesizing kenaf/phenol formaldehydes (PF), kenaf/fiberfrax hybrid PF and fiberfrax/PF composites and determined that 43% of the kenaf fiber was enough to optimize hardness, tensile strength, and flexural strength [105]. Likewise, Sosiati et al. synthesized sisal/poly-methyl methacrylate (PMMA) as a biomedical composite due to its high compatibility with human tissues, showing that a load of 30% of the fiber yielded optimal properties in dental and prosthesis applications [106].

Biocomposites are prepared using several manufacturing methods, such as injection molding, vacuum infusion, compression molding, resin transfer molding (RTM), hand layup, and direct extrusion [14,91,107]. These techniques have been widely used and updated by many researchers. However, some factors can affect the manufacturing process, such as humidity, temperature, pressure, and others [108].

#### 3.3.1. Injection Molding

The injection molding method is used mostly for the mass production of composites [109]. This process consists of injecting the materials into a mold to produce a biocomposite; this method allows us to produce different types of thermosetting or thermoplastic polymers. In a typical experiment, polymeric materials are mixed in a hot barrel, then forced out of the mixture through a mold cavity to be cooled and hardened to the cavity’s configurations. Modeling machines can be arranged vertically or horizontally. In general, the principle of manufacturing by injection molding consists of heating and injecting the materials into a mold [91,92]. An advantage of injection molding over direct extrusion is that it results in materials with three-dimensional shapes for various industrial applications [110]. For instance, Jamadon et al. carried out the synthesis of poly-lactic acid (PLA) reinforced with magnesium hydroxide (PLA/Mg(OH)_2_) using the injection molding method to be applied in bone implants [111].

#### 3.3.2. Resin Transfer Molding

In the RTM process, reinforcing fibers are placed in a mold cavity that is clamped and closed [112]. Subsequently, the polymeric resin mixture is injected into the mold cavity using pressure through single or multiple inlet ports until the mold is filled. In this case, the atmospheric pressure is lower than the pressure inside the hole. After cooling, the part is removed from the mold, and a post-cure is then needed to cure the resin [92,113]. Unlike hand layup, the biocomposite formed using RTM molding absorbs less moisture, owing to its limited porosity [114]. For example, Ravindran et al. prepared panels made with flax fiber and recyclates (flax/epoxy) exhibiting significant increases in the flexural modulus. The material characteristics gained from the resin transfer molding process are significantly better than those obtained via the other method [115]. These findings highlight the fact that RTM is a straightforward method to prepare fiber composites with excellent properties.

#### 3.3.3. Compression Molding

Manufacturing by compression molding is based on the production of compounds that have thermoplastic characteristics and are of light molecular weight [116]. First, the materials are placed in a previously heated open space (mold). Subsequently, this mold is closed, and a certain pressure is applied so that the polymeric materials acquire the shape of the mold and have uniform contact with the entire area. In the case of thermoplastic materials, the cooling process is critical and should be applied before being expelled from the mold [92]. The final product obtained by compression molding exhibits more homogeneous physical properties than those produced via injection molding. Furthermore, the fiber length is less affected using compression molding; therefore, the as-obtained composite will break less than when produced using injection molding, leading to improved mechanical properties compared to short fibers [117]. Sathish et al. developed the synthesis of two different composites using ramie fiber as a base combined either with hemp or coir fibers by compression molding [118]. This research showed that both composites can be used in the production of joints and bone fixtures to reduce pain in patients.

#### 3.3.4. Hand Layup

The hand layup is the oldest fabrication method used and consists of the manual manufacture of mold layers and the subsequent application of a resin matrix [119]. The compound is then crushed and rolled to distribute the placed resin evenly, eliminate the air that might be trapped, and obtain a better interaction between the reinforcement and the matrix [120]. This method mainly depends on the operator skills. In addition, this method cannot load much fiber, but longer fibers can be used. This method is quite attractive for its low cost [121]. For instance, Rao et al. prepared woven basalt fiber (55% fiber load) reinforced with Araldite LY556-Araudr HY951 prepared by hand layup and showed that this composite had excellent water absorption behavior and mechanical strengths, which could be used in biomedical applications [122].

#### 3.3.5. Direct Extrusion

Direct extrusion is the most common method for creating products with a constant cross-section [123]. This method generally consists of softening the matrix material, usually in the form of beads, and mixing it with a fiber bundle passed through an extruder that may have one or two screws [107,120]. Proper fiber dispersion is critical in achieving high material performance. Like the injection molding method, the design of the processing screws influences the morphology, final dimensions, and properties of the final fiber-reinforced material. A twin-screw extruder is recommended to achieve greater homogeneity and good fiber dispersion [107]. For example, direct extrusion has been widely used in the fabrication of different implants and prostheses because of their lightweight nature [124].

Table 4 discusses the advantages and disadvantages of the main techniques used to manufacture composites made from the plant fibers.

## 4. Plant Fiber Composites

### 4.1. Advantages in Comparison with other Synthetic/Glass Composites

Flax, jute, hemp, sisal, bamboo and kenaf are widely used in biocomposites [101]. Spinifex littorals fibers (SLF) have some applications as a reinforcement material to replace glass fibers [125]. It was determined that SLF has a 76.20 wt% of cellulose content and lignin. Therefore, SLF is appropriate for increasing composites’ resilience, biodegradability, and fire resistance. Mechanical tests on SLF composites showed that tensile, flexural, impact strength and hardness increased by adding up to 40 wt% of fiber content to exhibit specific properties that are comparable to those of glass fiber composites. Another study tested flax, jute, and banana fibers to replace glass fibers in an epoxy matrix [126]. As a result, flax fibers perform better than the ones from jute and banana at specific concentrations. In addition, flax increases flexural strength and Young’s modulus, which is appropriate for lightly loaded structures and could be used for biomedical applications [127].

Cellulosic/lignin fibers exert lower immunogenicity than their glass/synthetic counterparts, making, therefore, these natural fibers suitable for various biomedical applications, such as drug delivery [63,73], tissue engineering and regeneration [128], and cosmetics [129]. Furthermore, plant fibers are commonly used in medical textiles, diapers, and other applications [60]. For instance, Milanovic et al. reported the oxidation of hemp fibers with potassium permanganate to manufacture fine, soft materials with potential use in sports clothing [130]. It was also shown that silver nanoparticles on cotton fabric with curcumin (used as a carrier during the synthesis) reduce the cell toxicity of the composite [131]. It improves the fiber’s cell viability and broad-spectrum antimicrobial activity, along with its antioxidant properties for the management of chronic wounds. Another study demonstrated that a similar composite, made of curcumin-delivering silver nanoparticles attached to cotton fibers, has a suitable swelling capacity and biological and mechanical properties, and may find application as a wound dressing, since it improves the viability of L929 cells [132]. These studies support that cellulosic fibers can be used for in vivo applications as natural additives in a variety of composites [133].

Ranganathan et al. screened the effect of viscose fiber content on the mechanical properties of several composites containing given amounts of polypropylene (PP), maleated PP (MAPP, the compatibilizing agent), and jute, and manufactured via direct long fiber thermoplastic extrusion and compression molding [134]. Their results show that viscose fibers increase the energy absorption of the composite and, at the same time, decrease the heat deflection temperature (HDT) values. In addition, the addition of 2 wt% of MAPP improves the composite properties in general. Although similar applications of synthetic glass fibers have been reported, glass fibers need advanced surface modifications to achieve comparable purposes as natural ones do [135]. To that aim, Chen et al., for instance, designed a strategy to modify bulk metallic surfaces by electrophoretic deposition of phosphate glass fibers (PGF) aligned in a poly(acrylic acid) matrix [136]. Depending on PGF concentration and orientation, the newly obtained bioactive surface proved to be efficient in increasing the cell viability and enhancing the cell migration and differentiation, in addition to increasing gene expression. All these findings determine that PGFs have high potential for osteogenic differentiation.

### 4.2. Current Plant Fiber Composites 

To date, only a small portion of the existing natural fibers has been explored for potential uses in composites for structural and non-structural applications in the biomedical field [137,138]. However, the vast diversity of plant fibers may enable the isolation of fibers with excellent mechanical properties, such as tensile and flexural strength [139]. In addition, the unique properties of biocomposites may be tuned and tailored by several parameters, such as the fiber, matrix, filler, and processing methods [13]. Even though they offer key advantages, such as low immunogenicity and outstanding water absorption capability, some plant fibers still lack or display mediocre desired properties (e.g., thermal, or mechanical) [140]. Therefore, a suitable solution resides in combining natural fibers with synthetic materials to give rise to biocomposites that meet specific requirements that are critical to achieving the targeted application. Table 5 summarizes some concrete application examples of commonly used plant fibers explained in Figure 2 in combination with other synthetic/glass/natural fibers.

Most investigations on plant fiber composites use epoxy resins due to their mechanical, electrical, and chemical properties. While plant fibers increase flexibility and tensile strength, adding graphene improves the resins’ shear strength [141]. As additives, natural sources contribute to improving the composite stiffness, biocompatibility, and bioactivity [150]. For instance, Hong et al. used melt compounding extrusion to synthesize poly(ε-caprolactone) (PCL) nanocomposites with surface-oxidized cellulose nanocrystals (SO-CNCs) decorated with carboxyl groups [151]. The results showed that a concentration of 10 wt% of SO-CNCs doubles the original value of PCL Young’s modulus. In addition, the ultimate tensile strength and crystallization temperature are also favored. Based on its remarkable properties, the newly designed composite holds great promise in tissue engineering as bone scaffolds, since SO-CNCs induce the biomineralization of calcium phosphate.

### 4.3. Biomedical Applications of Natural Fiber-Reinforced Composites

#### 4.3.1. Drug Delivery and Antibiotic Applications

Investigations have focused on developing novel drug delivery systems to release pharmaceuticals more effectively and safely into the body [152]. In addition, these systems enable better control over the number of therapeutic drugs that should be released in a sustained manner [153]. Thus, many scaffolds based on natural fibers have been developed and implemented to encapsulate and deliver drugs [17,154]. For instance, the fibers produced using electrospinning allow the appropriate incorporation of the drugs [138]. Consequently, the drug-loading capacity increases to elicit a sustained release of drugs and/or of natural extracts/nutraceuticals [155]. Nonetheless, fibers present a limitation related to the lack of formation of 3D networks that can affect cell migration/infiltration [156]. To overcome this obstacle, the idea of combining natural fibers with other compounds and creating composites arises.

For instance, Macha et al. conducted an in vitro study on hand-woven cotton fabric/polylactic acid (PLA) composites and found that these composites are promising for the delivery of amoxicillin, which is an antibiotic widely used to stop bacteria growth and treat certain bacterial infections [157]. Another potential biocomposite found in the literature is the sericin fiber/poly(vinyl alcohol) composite loaded with tigecycline, an antibiotic commonly used to treat skin tissue bacterial infections [158]. This particular composite depicted a suitable morphology, porosity, and mechanical stability for drug delivery. Moreover, it presented strong antibacterial activity against *Escherichia coli* and *Bacillus subtilis*, accelerating the wound-healing process. Hence, natural fiber-based composites, combined with specific biomolecules, create the opportunity for biomedical applications, such as in drug delivery, antibiotic activity, and wound healing.

#### 4.3.2. Orthopedics and Prostheses

Current natural fiber-reinforced polymer composites are extensively studied to determine the feasibility of their application in orthopedics [159]. Natural fibers, such as those of sisal, flax, jute, or banana, can be potentially used to treat bone fracturing when properly combined with a specific polymer that can enhance the overall mechanical properties of the composite [101]. Chandramohan and Marimuthu found that the orientation or placement of the natural fibers determines whether the composite is isotropic or highly anisotropic [160]. Moreover, they discovered that sisal depicted the best mechanical properties; nonetheless, roselle fiber exhibited more potential for internal and external fixation of fractured human bones if combined with calcium phosphate and hydroxyapatite (hybrid) composites. Another example of a promising hybrid composite material consists of 15% flax and 15% ramie with an underlying bio-epoxy resin matrix. The results show that the mechanical properties of these hybrid composites are comparable to the femur and tibia bone, which indicates their suitability in orthopedic implant applications. Moreover, composites with an underlying biopolymer matrix are used to develop lower-limb prostheses raising the hope for the design of their upper limb analogs [161].

Composites reinforced with natural fibers have been reported as a potential material for prosthetics, in addition to those with an underlying biopolymer matrix [162]. For instance, Hamad et al. studied the mechanical properties of laminated composites for prosthetic sockets, which were prepared using the vacuum bagging technique and reinforced with natural fibers, such as jute, combined with glass, carbon and perlon, and bonded within a polyester resin matrix [163]. This study shows that the mechanical properties of the composites are influenced by the type and number of reinforcing layers, and the best composite consisted of three layers of jute and four layers of carbon fibers, resulting in a tensile strength and modulus of elasticity of 162 MPa and 3.60 GPa, respectively.

These studies demonstrate that certain combinations of natural fibers and polymers can significantly improve the mechanical properties of composite materials. Therefore, natural fibers can be a promising and more sustainable alternative to conventional materials used in the manufacturing of prostheses and orthopedic devices.

#### 4.3.3. Bone Tissue Engineering

Natural fiber composites can be used for biomedical applications for bone and tissue repair and reconstruction [164]. Natural fiber reinforcing materials are embedded in a biopolymer matrix as a dispersed phase to improve the stiffness and strength of the biocomposites by carrying the applied stress and load [165]. Natural fibers, such as coir, ramie, flax, silk, and jute, have been utilized for long-time reinforcements in biocomposite scaffolds [15]. In addition, more natural fibers such as hemp, kenaf, bamboo, banana, sisal, wheat, sugarcane, oil palm, cotton, and coconut have been gaining attention for manufacturing bone tissue engineering scaffolds. Agricultural wastes, such as almond shells, sugarcane residues, and walnut waste, are used as a source of fibers in various biopolymers matrices to produce environmentally friendly, more affordable, and highly reinforced scaffolds [166]. The mechanical properties of natural fiber biocomposites, such as tensile strength, strongly depend on the type of fiber.

#### 4.3.4. Nanotechnology

By integrating nanotechnology with natural plant fibers, researchers have sought to enhance their biomedical properties and expand their applications in the healthcare domain. Several studies have improved engineered biocomposites’ mechanical and tribological properties with inorganic nanoparticles such as ZrO_2_, ZnO, CuS, and TiO_2_ into polymer matrices [167]. The advantages of the incorporation of these nanoparticles in the composites enhance their thermal stability and reduce their water absorption capacity. The resultant hybrid materials exhibit improved biocompatibility, mechanical strength, controlled release capabilities, and bioactive functionalities by incorporating nanoparticles, nanofibers, and nanocomposites. For instance, Vasconcelos et al. successfully demonstrate the deposition of TiO_2_ nanocoatings on ginger lily fibers using DC reactive magnetron sputtering. The research provides valuable insights into the morphological, mechanical, optical, and photocatalytic properties of the TiO_2_-coated fibers, opening up possibilities for their utilization in various fields, including textiles and composites in the medical area [168].

The fabrication of nanofibers starting from natural plant polymers, such as cellulose, chitosan, and silk relies on various techniques, such as electrospinning or self-assembly methods [169]. These nanofibers exhibit high surface area-to-volume ratios, enabling enhanced drug-loading capacities and controlled release profiles for targeted drug delivery applications. Orasugh et al. developed a jute cellulose nano-fibrils/hydroxypropylmethylcellulose nanocomposite as a novel material with potential for application in transdermal drug delivery systems [63]. The nanocomposite offers improved mechanical strength, flexibility, thermal stability, water uptake capacity, controlled drug release, and excellent skin adhesion properties.

Moreover, incorporating nanoparticles, such as silver, gold, or magnetic nanoparticles, into natural plant fiber matrices has shown great potential in imparting antibacterial, antimicrobial, or magnetic properties to the resulting nanocomposites [170]. These hybrid materials exhibit improved wound healing capabilities, antimicrobial effects, and diagnostic functionalities.

### 4.4. Limitations of Composites Reinforced Using Plant Fibers

Plant fibers present various issues related to inadequate interfacial adhesion, high levels of moisture absorption, poor wettability, poor fire resistance, and low impact strength and durability [91]. As a result, fiber plant composites have innate hydrophilicity and flammability. Depending on the target application, these drawbacks may restrict the use of plant fibers as polymer reinforcement [171]. Moreover, physical, and chemical modifications are necessary to make them compatible with the matrix, while conserving the unique properties of fibers [172,173]. The raw composition plays a determining role in the final characteristics of natural fibers [174]. Their extraction by alkaline treatments allows the removal of non-cellulosic components, such as lignin, hemicellulose, pectin, and waxes, and processes the fiber surface roughness for a better interlink between the fibers and the embedding matrix [175]. Vinyl ester, polyester, epoxy, poly(lactic acid), and polypropylene are used as a matrix for cellulosic fibers resulting in high-profit composites [176].

In biomedical applications, it is necessary to consider factors such as bioaccumulation, biodegradation, excretion, and the effect of enzymes, hormones, and the immune response on the fibers. Lignin, for example, possesses excellent antioxidant, antimicrobial, and optical properties, making it suitable for biomedical applications, although some challenges are yet to be overcome [177]. Typically, its overall molecular weight and complex structures limit its utilization for scale-up applications. To address these hurdles and promote the continuous development of lignin-based materials, cooperation between materials scientists, biomedical engineers and public health researchers is required. For instance, Domínguez-Robles et al. combined lignin and polybutylene succinate (PBS) by hot melt extrusion to obtain biocomposites exhibiting antimicrobial and antioxidant properties [32]. Although the material’s density, Young’s modulus, and tensile strength remained almost unchanged, its antioxidant effect was evident since the novel biocomposite reduced up to 80% of the DPPH (2,2-diphenyl-1-picrylhydrazyl) initial concentration. These findings are another example of fiber hybridization in composites whose properties mainly depend on fiber type, length and orientation, bonding to the matrix, and general arrangement [178]. By tailoring their properties, hybrid composites constitute a special class of biomaterials that may surpass their non-hybrid analogs in biomedical applications.

## 5. Conclusions

Many industries rely on developing composites to obtain novel, environmentally friendly materials with tunable properties for the target application. Natural fibers, mainly made of cellulose, have been widely studied as a critical component of composites. These fibers provide the composite with unique properties, such as enhanced mechanical resilience, flexibility, biocompatibility, and antimicrobial effects. In addition, they are highly hydrophilic, biocompatible, and biodegradable, and exhibit low immunogenicity. Most often, they should be combined or hybridized with other natural or glass/synthetic fibers to achieve good mechanical properties. In biomedicine, biocomposites have received particular attention since natural fibers can enhance the composite immune acceptance and improve the cell viability, as evidenced by in vitro studies for various potential biomedical applications, including prostheses, drug delivery technologies, wound healing, and scaffolds for tissue engineering.

Some parameters, such as the source of the natural fiber, cellulose content, extraction, and preparation methods, affect the final properties of the biocomposites. Additionally, the preparation method should be carefully chosen depending on the desired definitive characteristics. Manufacturing processes can damage the biocomposites if humidity, pressure, and synthesis temperature are not meticulously controlled. Consequently, investigating manufacturing techniques is a crucial step in the development of composites to enhance or decrease their tensile, flexural, and impact strength towards, ultimately, their translation from the lab bench to commercial products. Finally, nanomaterials, carbon-based materials and bioactive compounds should be considered for future investigations to face the encountered challenges and limitations of hybrid composites based on natural fibers.

## Figures and Tables

**Table 1:** Examples of common natural fibers for biomedical applications.

Natural Fiber	Subproduct	Properties	Assay	Potential Biomedical Application	Refs.
Jute	Cellulose nanowhiskers extracted from TEMPO-oxidized jute fibers	Ultrathin diameters and high crystallinity (69.72%), high yield (over 80%) and high surface area	In vitro	Nanowhiskers with smaller widths would be particularly useful for applications as a reinforcing phase in the nanocomposites, as well as in tissue engineering and pharmaceutical additives.	[62]
	Cellulose nano-fibrils (CNF) derived from raw jute fibers	High surface area, good rheological properties, promising water absorption, non-toxicity	In vitro	Excellent candidate for transdermal drug delivery system because the cumulative drug release percentage is decreased with the increase in the CNF concentration in the bionanocomposite film.	[63]
Flax	Flax fibers enriched with poly-β-hydroxybutyrate (PHB)	Higher average resistance related to tensile assay and improvement of elastic properties, biocompatibility, non-immunogenicity	Preclinical	Biodegradable and biocompatible polymers useful in the fabrication of new dressing for chronic wounds with successful preclinical trial.	[64]
	Flax textiles for blood-contacting applications	Flax textiles uniquely combine hydrophilicity and strength, hydrophilic material	In vitro	Albumin coating on flax fibers reduces thrombogenicity; this can be used for implantable devices.	[65]
	Implantable mesh structures in surgery	Non-biodegradability, good physical properties	In vitro and in vivo	Used for incisional hernias of the abdominal wall after removing endotoxins in flax fiber.	[66]
Ramie	Application as surgical suture biomaterial	Excellent biocompatibility, tensile strength, and wound closure efficacy	In vitro and in vivo	Novel, cost-effective biomaterial with efficient healing properties of superficial wounds for suture material applica-tion.	[67]
	Cellulose nanocrystals isolated from ramie fibers	High crystallinity and improved thermal stability	-	Potential application as reinforcing fillers in nanocomposites.	[68]
Kenaf	Biomimetic hydroxyapatite growth in kenaf fiber	Good mechanical properties, biodegradability, enhanced adhesion of osteoblast cells to cellulose surface	-	The coating on kenaf fibers can be applied to bone tissue engineering.	[69]
	Mixed natural fibers with polymers	Flexural strength enhancement and shore hardness	-	Biomedical orthopedic application in fracture or tissue replacement.	[70]
Sisal	Sulfonated cellulose nanowhiskers extracted from fibers	Excellent biocompatibility and biodegradability	-	Potential use in tissue engineering, cosmetics, and drug delivery.	[71]
	Microcrystalline cellulose prepared from sisal fibers	Good crystallinity and shape as long thread-like fibers	In vitro	Immediate release as well as sustained release in oral solid dosage forms.	[72]
Banana	Porous microcrystalline cellulose extracted from pseudostem fibers	Highly crystalline, rod-shaped, and non-aggregating properties	In vitro	Capability to sustainably disperse isoniazid medicine, which is used for the treatment of anti-tuberculosis at regular time intervals.	[73]
	Cellulose nanofibers isolated from banana fibers	Small size and high crystallinity	-	It can be used as a promising reinforcing material in a polymer matrix to further enhance the properties and, in return, extend its applicability in pharmaceuticals, bio-nanocomposite, and tissue engineering.	[74]

**Table 2:** Mechanical property requirements of fibers based on biomedical applications.

Strength	Definition	Biomedical Material Classes	Refs.
Tensile strength	It is determined as the material’s capacity to resist forces applied in the longitudinal axis.	Co-Cr, Ti-alloys and stainless steel are materials with high tensile strength, while ceramic and polymer biomaterials exhibit reduced strength. Therefore, natural biopolymers must fulfill a maximum or optimum percentage of elongation, Young’s modulus, yield, and ultimate tensile strength regarding the long-term use of biomaterials.	[80]
Flexural strength	It is defined as the material’s ability to resist the deformation under load.	Zirconia-based ceramics are commonly used in restorative dentistry due to their excellent esthetics and biocompatibility properties. The flexural strength of these materials is a crucial mechanical property that determines their ability to withstand occlusal forces and resist fractures. Flexural Young’s modulus, flexural loading, and strength parameters should be studied for the natural polymer as a biomaterial candidate.	[81]
Impact strength	It is evaluated by four failure modes and analyzes the toughness and notch sensitivity.	These properties influence the product’s safety in use as well as its liability. Impacted properties are related to the service life and performance of the product.	[82]
Thermal strength	The ability of the fiber to withstand high temperatures without performance failure	In natural fibers, hemicellulose, cellulose, and pectin are sensitive to different temperature ranges. Therefore, they may be altered by chemical or physical processes.	[83]

**Table 3:** Mechanical properties and content of plant fibers.

Plant	Cellulose (%)	Hemicellulose (%)	Lignin (%)	Strength (MPa)	Elongation (%)	Young’s Modulus (GPa)	Refs.
Cotton	90–95	2–3	0.2–0.5	287–587	7–8	5–13	[58]
Sisal	65	12	10	611–637	2–25	9.4–22	[10]
Banana	60–65	6–8	5–10	529–914	3–10	17–32	[10,93]
Kenaf	50–57	22	10	240–930	1.6	14–53	[94]
Hemp	57–77	14–22.4	3.7–13	690	1–3.5	-	[95]
Bagasse	55.2	17	25	290	-	17	[10,90]
Jute	61–71	14–20	12–13	393–773	1.5–1.8	10–30	[94,96]
Flax	67–71	18–20	3	343–1035	1.2–3	27.6–160	[10,58]
Pineapple	70–80	18.8	12.7	126.6	2.2	4.4	[93,96]
Bamboo	74	13	10	391–100	2	11–30	[93]

**Table 4:** Advantages and disadvantages of manufacturing techniques of plant fiber composites.

Manufacturing Techniques	Advantages	Disadvantages
Injection molding	- Low labor cost - Material and color flexibility - Fast production	- Part design restriction - High initial tooling and machinery cost
RTM molding	- Low material wastage - Uniform thickness of composite part	- High tooling cost - Complex mold design and limited size
Compression molding	- Low cost at large production scale - High surface quality	- High equipment cost - Not suitable for structural parts
Hand layup	- Simple principles - Versatility - Low cost	- Labor intensiveness - Low viscosity resin
Extrusion	- Fast and economical method - Dimensional repeatability	- Limited part size - Limited to constant cross-section parts

**Table 5:** Examples of composites made by combining widespread natural fibers and synthetic materials, their improved properties, and potential applications.

Source	Scientific Name	Composite Composition	Property/Application	Refs.
Jute	*Corchorus capsularis*	Graphene-based natural jute fiber in an epoxy matrix	Stiffness-driven applications	[141]
Flax	*Linum usitatissimum*	Flax fibers pre-impregnated with fire-retardant epoxy polymer	Increased flexural properties and water absorption	[142]
Kenaf	*Hibiscus cannabinus* L	Kenaf fiber-reinforced epoxy matrix	Enhanced thermal stability for applications requiring superior performance	[143]
Hemp	*Cannabis sativa* ssp. *sativa*	Ramie fibers and hemp fibers in polypropylene resin	Enhanced tensile strength, Young’s modulus, and density	[118]
Cotton	*Gossypium*	3-aminopropyltriethoxysilane (APTS)-functionalized cotton fibers electrostatically interacting with silver nanowires and reduced graphene oxide	Enhanced fatigue and anti-bending properties at cryogenic temperature	[144]
Sisal	*Agave sisalana*	Unsaturated polyester resin, hardener, and sisal fibers	High specific strength, lightweight, and biodegradability for automotive industry	[145]
Banana	*Musa*	Glass/banana fibers in epoxy resin	Development of lightweight structural materials	[146]
Wood	*-*	Wood fiber, polypropylene, glass, and carbon fibers	Enhanced tensile strength and modulus, flame-retarding properties	[147]
Sugarcane	*Saccharum officinarum*	Sugar cane fibers and powdered rice husk	Decreased tensile and yield strength, and ductility	[148]
Bamboo	*Bambusa*	Bamboo fibers and epoxy resin	Tensile strength and Young’s modulus of the composite increase as the bamboo fiber diameter decreases	[149]

## Data Availability

Not applicable.
